# Umbilical Cord Blood Mononuclear Cell Treatment for Neonatal Rats With Hypoxic Ischemia

**DOI:** 10.3389/fncel.2022.823320

**Published:** 2022-03-02

**Authors:** Hao Lyu, Dong Ming Sun, Chi Ping Ng, Wendy S. Cheng, Jun Fan Chen, Yu Zhong He, Sin Yu Lam, Zhi Yuan Zheng, Guo Dong Huang, Chi Chiu Wang, Wise Young, Wai Sang Poon

**Affiliations:** ^1^Division of Neurosurgery, Department of Surgery, Faculty of Medicine, The Chinese University of Hong Kong, Shatin, Hong Kong SAR, China; ^2^Shenzhen Key Laboratory of Neurosurgery, Department of Neurosurgery, The Shenzhen Second People’s Hospital, First Affiliated Hospital of Shenzhen University, Shenzhen, China; ^3^W. M. Keck Center for Collaborative Neuroscience, Rutgers, The State University of New Jersey, Piscataway, NJ, United States; ^4^Mononuclear Therapeutics Limited, Hong Kong, Hong Kong SAR, China; ^5^Department of Neurosurgery, Hainan Hospital of People’s Liberation Army General Hospital, Sanya, China; ^6^Department of Obstetrics and Gynecology, Li Ka Shing Institute of Health Sciences, School of Biomedical Sciences, Chinese University of Hong Kong-Sichuan University Joint Laboratory in Reproductive Medicine, Shatin, Hong Kong SAR, China

**Keywords:** hypoxic ischemia encephalopathy (HIE), animal model, cell therapeutic, umbilical cord blood (UCB), mononucleal cells

## Abstract

**Background:**

Hypoxic-ischemic encephalopathy (HIE) occurs when an infant’s brain has not received adequate oxygen and blood supply, resulting in ischemic and hypoxic damage. Currently, supportive care and hypothermia therapy have been the standard treatment for HIE. However, there are still over 20% of treated infants died and 19–30% survived with significant disability. HIE animal model was first established by Rice et al., involving the ligation of one common carotid artery followed by hypoxia. In this study, we investigated human umbilical cord blood (HUCB) and its two components mononuclear cell (MNC) and red cell fraction (RCF) in both short and long term study using a modified HIE rat model.

**Methods:**

In this modified HIE model, both common carotid arteries were occluded, breathing 8% oxygen in a hypoxic chamber for 60-min, followed by the release of the common carotid arteries ligature, mimicking reperfusion injury. For cell therapeutic study, cells were intravenously injected to HIE rat pups, and both behavioral and histological changes were assessed at selected time points.

**Result:**

Statistically significant behavioral improvements were demonstrated on Day 7 and 1 month between saline treated HIE rats and UCB/MNC treated rats. However, at 3 months, the therapeutic improvements were only showed between saline treated HIE animals and MNC treated HIE rats. For histological analysis 1 month after cell injection, the number of functional neurons were statistically increased between saline treated HIE and UCB/MNC/RCF treated HIE rats. At 3 months, the significant increase in functional neurons was only present in MNC treated HIE rats.

**Conclusion:**

We have used a bilateral temporary occlusion of 60 min, a moderately brain damaged model, for cell therapeutic studies. HUCB mononuclear cell (MNC) therapy showed benefits in neonatal HIE rats in both short and long term behavioral and histological assessments.

## Introduction

Hypoxic-ischemic encephalopathy (HIE) occurs when the infant brain is deprived of oxygen due to limited blood flow. It is a major cause of neurological deficits in new-born babies. The incidence of HIE is approximately 5 per 1000 live births worldwide (Kurinczuk et al., 2010). It is characterized by edema, secondary cellular energy failure and excitotoxicity with a major mitochondrial dysfunction, which triggers cell death and clinical complications of moderate to severe damage in the brains of the new-born (Gunn et al., 1997; Bennet et al., 2012). Of the neonates who survive the HIE, 20% develop cerebral palsy (CP) (Lehnardt et al., 2003; Gluckman and Rocha, 2004; Gluckman et al., 2005; Eklind et al., 2005; Shankaran et al., 2005; Jacobs et al., 2011).

Immediate supportive care is of paramount importance in the resuscitation of these HIE babies by restoring sufficient cerebral blood flow (CBF), maintaining adequate oxygen and glucose supply to the brain, and finally preventing further brain injury (Douglas-Escobar and Weiss, 2015). Hypothermia therapy has been used for term infants with HIE. This treatment involves decreasing the neonate’s body temperature to 33–35°C, and has been proven to reduce the risks of neurodevelopmental deficits and death in a long-term study of neonates with moderate to severe HIE (Shankaran et al., 2005; Gunn et al., 2008; Azzopardi et al., 2009).

Despite receiving the best medical care, including hypothermia therapy, approximately 20% of infants with HIE die or survive with moderate to severe neurological disabilities, such as CP (Shankaran et al., 2005; Graham et al., 2016). Hypothermia therapy is effective only when administered within 6 h after birth (Shankaran et al., 2005; Higgins et al., 2011). Therefore, novel therapeutic approaches, especially those with a long therapeutic window, are warranted for infants with HIE.

Cell therapy has received increasing attention in the past two decades, not only for its regenerative ability but also for its therapeutic window (Jantzie et al., 2018). Human umbilical cord blood (HUCB) consists of a large amount of monocytes (40%), lymphocytes (40%), neutrophils (10%), and the remaining 10%, a mixture of endothelial progenitor cells, multipotent stem cells, as well as the mesenchymal stem cells (MSCs), similar to the bone marrow cell population (Jaing, 2014). In the literature, these MNCs in HUCB have been reported to consist of 3–10% immature progenitor cells (Jaing, 2014; Zhu et al., 2016). Moreover, it can be readily collected and stored for future therapy and research. The immune rejection risk is low in HUCB transplantation as its HLA antigen levels remain low in comparison with cells harvested from adults, such as bone marrow derived MSCs (Penny et al., 2019). HUCB was first used in animal models of HIE by Meier and her colleagues. Ten million HUCB MNCs in 0.5 ml was delivered intraperitoneally into the postnatal day 7 rat pups 24 h after HIE. The Meier study has demonstrated the presence of MNCs in the affected cerebral hemispheres of the treated HIE pups, without neuronal or astrocytic differentiation (Meier et al., 2006). Another study found that intravenous injection of a small dose of HUCB MNCs (15,000) 7 days after HIE, assisted by blood brain barrier manipulation with intravenous mannitol, improved motor coordination of these MNCs-treated rat pups (Yasuhara et al., 2010). A clinical research group at Duke University tested the feasibility and safety of transplanting autologous HUCB in neonates with HIE. The efficacy of the cell therapy was assessed based on neurological development scores until the recruited infants were 1-year-old (Pimentel-Coelho and Mendez-Otero, 2010; Cotten et al., 2014).

However, several aspects of HUCB as a treatment for HIE remain unclear, including the mechanisms of its therapeutic effects, the cell types that contribute the most to this effect and the optimal cell transplantation route, number of cells and timing of transplantation. Therefore, in this study, we investigated the effects of transplantation with total HUCB and its two components, mononuclear cells (MNCs) and the red cell fraction (RCF), over the short term (7 days after cell transplantation) and long term (1 and 3 months after cell transplantation) in a modified HIE rat model (Lyu et al., 2021).

## Materials and Methods

### Human Umbilical Cord Blood, Mononuclear Cell, and Red Cell Fraction Preparation

The collection of Umbilical Cord Blood (UCB) was approved by the Joint Chinese University of Hong Kong-New Territories East Cluster Clinical Research Ethics Committee (CREC Reference Number 2016.250). Blood was collected from the umbilical cord and connected placenta from consented mothers in the Department of Obstetrics and Gynecology, The Prince of Wales Hospital, The Chinese University of Hong Kong. The mother has provided signed consent for the use of these materials. The blood was collected in bags containing anticoagulant and will be stored in liquid nitrogen if no immediate clinical or research requests were made. HUCB, MNC, and the RCF were prepared by density-gradient centrifugation based on laboratory protocols. The cells were re-suspended in 0.9% sodium chloride, and their cell numbers were determined. The cell viabilities were confirmed to be higher than 90%. The qualified collections of HUCB were processed by our collaborator Mononuclear Therapeutics Ltd (MT, Hong Kong SAR, China) using the latest SynGeneX^®^-1000 system and CryoPRO-5 Processing and Storage Bag (SynGen Inc, San Carlo, CA, United States).

### The Modified Hypoxic-Ischemic Encephalopathy Animal Model

Hypoxic-ischemic encephalopathy animal model was first established by Rice et al. (1981), and has been used extensively to explore the mechanisms of brain damage resulting from HIE and to test the effectiveness of potential therapeutic interventions. The HIE model involves the ligation of one common carotid artery followed by hypoxia (8% oxygen), however, this model did not simulate the real situation of human HIE. In this study, we used a modified bilateral temporary occlusion HIE animal model as we described recently, which better mimics the human situation of a difficult labor (Lyu et al., 2021). All animal experimentation protocols followed the guidelines for the care and use of animals and were approved by the ethics committee of the Chinese University of Hong Kong. The modified bilateral HIE model was used to induce the brain damage from hypoxia and ischemia in the 7-day-old neonatal rats. All animals were housed under a 12-h light/12-h dark cycle with free access to food and water. The rats were fully anesthetized with isoflurane (3–4% for induction and 1–2% for maintenance), and a small incision was made in the middle of the neck using an #11 scalpel. Using blunt dissection, the connective tissue and muscles were separated to clearly visualize blood vessels under an operating microscope. To establish the bilateral HIE model, both common carotid arteries (CCA) were temporally ligated with 6-0 silk suture. After the incision was closed with a suture, the pups were placed in a hypoxia chamber containing 8% oxygen balanced with 92% nitrogen for 60 min. A heating pad was used to maintain the pups’ body temperature in the chamber. At the end of hypoxia exposure, the pups were removed from the chamber and anesthetized again with isoflurane. Their incisions were opened, and the ligatures on both CCAs were released. The incisions were then closed with 6-0 sutures, and the pups were allowed to wake up in a recovery box. Buprenorphine (Buprenex) (0.05 mg/kg) was administered to the pups for 6 h for postoperative analgesia.

### Experimental Grouping

The animals were assigned randomly to six experimental groups: Normal control (no brain injury), HIE only, HIE with sham injection (with 0.9% sodium chloride), and HIE with cell therapy (HUCB, MNC or the RCF).

Animals in the HIE with sham injection group were intravenously injected with 200 μL of 0.9% sodium chloride 24 h after HIE modeling (i.e., bilateral CCA occlusion followed by hypoxia). In the cell therapy group, the rat pups were intravenously injected with 1 × 10^7^ of UCB, MNC or RCF in the selected volume of 200 μL or 0.2 ml, 24 h after HIE modeling.

### Cell Transplantation

For cell transplantation, the rat pups in the cell therapy group were fully anesthetized with isoflurane (3–4% for induction and 1–2% for maintenance), and a small incision was made in the left side of the neck using an #11 scalpel. Using blunt dissection, the connective tissue and muscles were separated to clearly visualized the left external jugular vein under a microscope. Using a 30G insulin syringe, 1 × 10^7^ cells (200 μL or 0.2 ml) were slowly injected in the direction of the heart over a period of 10 min to avoid complications due to fluid overload. Subsequently, the syringe was withdrawn, compression was applied to the wound for 5 min to stop bleeding. The incision was closed with one suture.

### Real-Time PCR

Human Alu Yb8 was detected 2 days after cell transplantation to determine the migration of HUCB and MNC in the pups’ brains. The rat pups were anesthetized with ketamine (75 mg/kg) and xylazine (5 mg/kg) and subjected to transcardiac perfusion with 0.9% sodium chloride followed by RNA later (Invitrogen™ RNAlater™ Stabilization Solution, AM7021). Total DNA was extracted from the brain, heart, liver, lungs, stomach, spleen and kidneys using a DNA extraction kit (Qiagen 69506) according to the manufacturer’s instructions. The standard curve was prepared. The primer set for the human Alu Yb8 sequence (sense 5′-CGAGGCGGGTGGATCATGAGGT-3′; antisense 5′-TCTGTCGCCCAGGCCGGACT-3′) was provided by our collaborator at Rutgers University (United States). Real-time PCR was performed with 300 ng of DNA and PowerUp SYBR Green Master Mix (Thermo Fisher, New York, NY, United States, A25741) using the QuantStudio™ 12K Flex System under the following conditions: 50°C for 2 min and 95°C for 10 min, followed by 40 cycles of 95°C for 15 s, 62°C for 15 s and 72°C for 30 s. The PCR data were analyzed using the Thermo Fisher online system^1^.

### Behavioral Assessments

The colleagues who conducted the behavioral tests were all blinded to the animal experimentation grouping.

#### Short-Term Assessments

##### Negative Geotaxis

To investigate the short-term effects of cell therapy on HIE, the negative geotaxis of the rat pups in all groups was tested on day 7 after cell transplantation. Briefly, the pups were placed on an inclined board (40°) with their head in the downward position. The time taken by the pups to turn 180° was recorded up to a maximum cut-off time of 60 s; i.e., the time to rotate for the pups that failed to rotate was recorded as 60 s.

#### Long-Term Assessments

To investigate the long-term effects of cell therapy, the pups in all groups were subjected to the beam balance test to assess motor function assessment and the Morris water maze test to assess spatial learning memory at 1 and 3 months after cell transplantation.

##### Beam Balance

For the beam balance test, a 100-cm-long, 1.75-cm-wide beam was set 90 cm above the floor. The test was conducted in an empty cage, and bedding was placed under the beam to protect the pups from injury if they fell. On the testing day, the rat pups were placed on the beam for 1 min, and the duration on the beam and balance score of each pup were recorded. Scoring was determined according to the following scale: (1) a stable balance posture; (2) a shaky posture indicated by grasping of the beam; (3) a balancing attempt by hanging on the beam; (4) falling off the beam after 10 s; (5) balancing attempt by hanging on the beam, followed by falling off the beam in 10 s; and (6) falling off the beam without hanging on. Each rat pup was tested three times, with a resting interval of a 5 min between each attempt.

##### Morris Water Maze

As the hippocampus was affected first after HIE, we assessed spatial-learning memory which correlated to hippocampus function. For this test, a 200-cm-diameter maze and a 10-cm-diameter escape platform were used, with four visual direction symbols placed as cues on the four tank walls. The water was maintained at room temperature. A camera connected to a computer was secured on the tank to record the pups’ swimming routes. The water tank was divided into four quadrants labeled as northeast (NE), southeast (SE), southwest (SW), and northwest (NW), and the escape platform was placed in the NW quadrant. Each rat pup was tested in four trials; the pup was placed facing the tank wall in a different quadrant in each trial, starting clockwise from NE to NW. The time taken by the pup to find the escape island in each trial (from each starting quadrant) was recorded. The cut-off time to reach the island was set at 120 s, and a 15-s interval was allowed between each trial (Vorhees and Williams, 2006). For this trial, the escape island was removed, and the animal was placed facing the tank wall at a new start position, viz. in the quadrant (SE quadrant) opposite to that in which the island was originally located (NW quadrant). The cut-off time to complete the trial was set at 60 s. A new start position in the probe trial ensures that the testing animal’s spatial preference is a reflection of the memory of the goal location, rather than a preference for a specific swimming path. The cumulative distance (distance between the pup and the center of the island at every 200 ms) and island crossing (number of times the pup entered the island location) were recorded to evaluate the pups’ reference memory.

### Immunohistochemistry

After behavioral assessments on day 7 and at 1 and 3 months, the rat pups were anesthetized by ketamine (75 mg/kg) and xylazine (5 mg/kg) and then subjected to transcardiac perfusion with 0.9% sodium chloride followed by 4% paraformaldehyde (PFA). The brains were removed, stored in 4% PFA for 24 h and then dehydrated in an alcohol gradient (50, 70, 90, and 100%). The dehydrated brains were embedded in paraffin, and 5-μm-thick sections were cut.

For the immunohistochemistry analysis, paraffin-embedded sections were stained with anti-NeuN primary antibody (Millipore, Burlington, MA, United States, MAB377, 1:200 dilution) at 4°C overnight then incubated with a secondary antibody (Abcam, Cambridge, United Kingdom, ab97023, Goat Anti-Mouse IgG, HRP, 1:500) for 2 h at room temperature. Subsequently, the sections were washed with PBS two times and then stained with 3,3′-diaminobenzidine for immunohistochemical analysis. Positive cell signals were recorded using an imaging microscope (Nikon Fi-3). For the quantitative analysis of NeuN-positive cells, six 0.5-mm^2^ squares in motor cortex were selected (three continuous slides per rat) and NeuN-positive cells were counted using Nikon NIS Element BR software.

### Statistical Analysis

GraphPad Prism 8.3.0 software was used for statistical analysis and graphics production. A one-way ANOVA with Tukey’s multiple comparison test was performed to assess the results of the behavioral tests and immunohistochemistry analysis. A *p*-value of <0.05 was considered significant to reject the null hypothesis. Normal distribution was checked by Kolmogorov–Smirnov test, a *p*-value of > 0.1was considered pass the normality test.

## Results

### Animal Sex, Number, Mortality and Grouping

The neonatal rats were un-sexed chosen, of which 42% were female. The mortality of HIE was 30% which is consist with our previous study (Lyu et al., 2021), and similar with the human situation (Shankaran et al., 2005). A total of 230 animals survived the HIE. Each neonatal rat was assigned randomly to six experimental groups: Normal control (*n* = 12 per group), HIE only (*n* = 12 per group), HIE with sham injection (transplanted with 0.9% sodium chloride, *n* = 12 per group), and HIE with cell therapy (HUCB, MNC or the RCF, *n* = 12 per group). The mortality of transplantation was 10%. Animals with too much bleeding during HIE surgery, intravenous injection, as well as blood clot found on section slides were excluded.

### Expression of Human Alu Yb8

**FIGURE 1:**
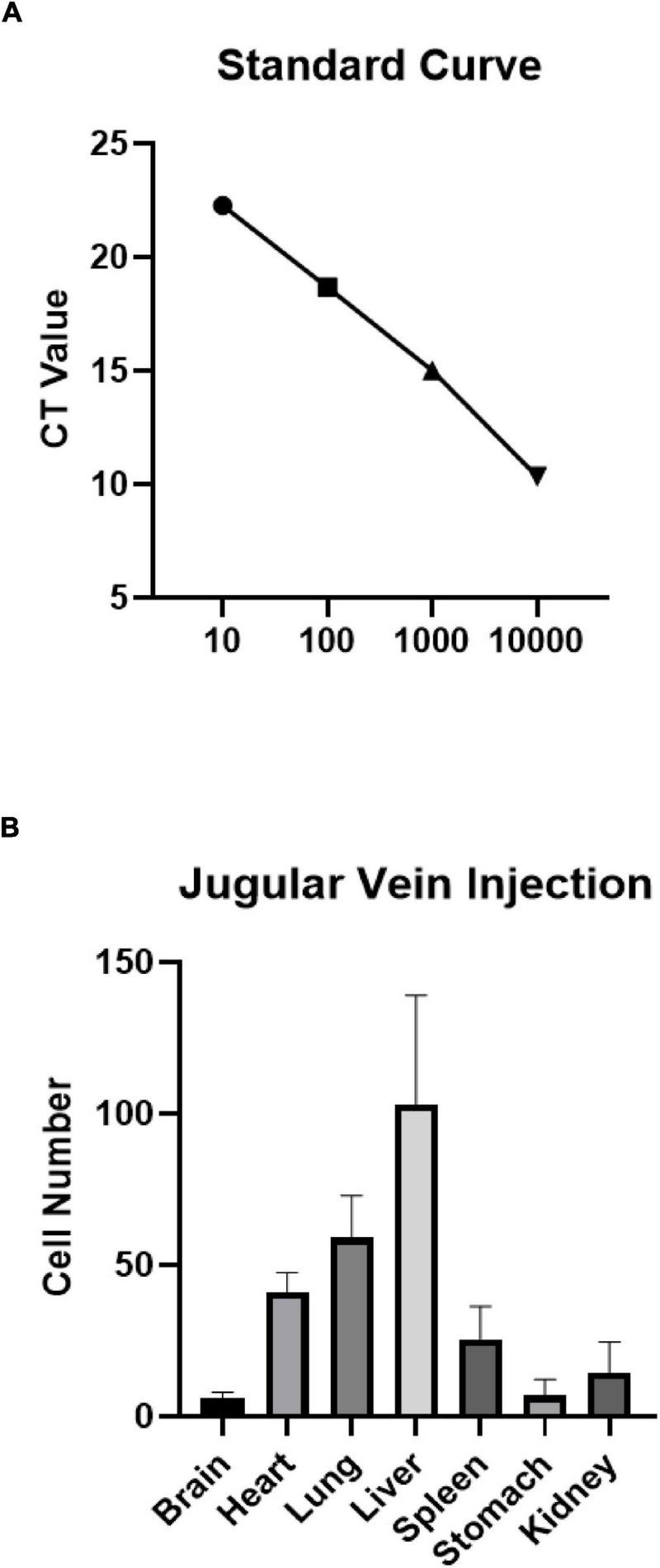
**(A)** Standard curve and **(B)** expression of human Alu Yb8 in selected organs based on real-time PCR results (*n* = 3 rat pups). The liver showed the highest number of injected MNCs, followed by the lungs and heart. Several cells were also detected in brain tissue.

Human UCB-derived MNC (1 × 10^7^) were injected intravenously into the rat pups 24 h after HIE modeling. The Alu Yb8 marker specific to these cells was detected in several organs, including the brains of the pups (*n* = 3). A standard curve was generated to estimate the number of cells based on the cycles that reach to plateau phase (Figure 1A). The results showed that human Alu Yb8 was detectable in all of the selected organs 2 days after intravenous cell injection, indicating that the transplanted human cells penetrated the blood-brain barrier and entered the damaged brain. Furthermore, the cell number estimates for various organs showed that the liver harvested the highest number of MNCs, followed by the lungs and heart, spleen, kidneys, stomach and brain (Figure 1).

### Negative Geotaxis

**FIGURE 2:**
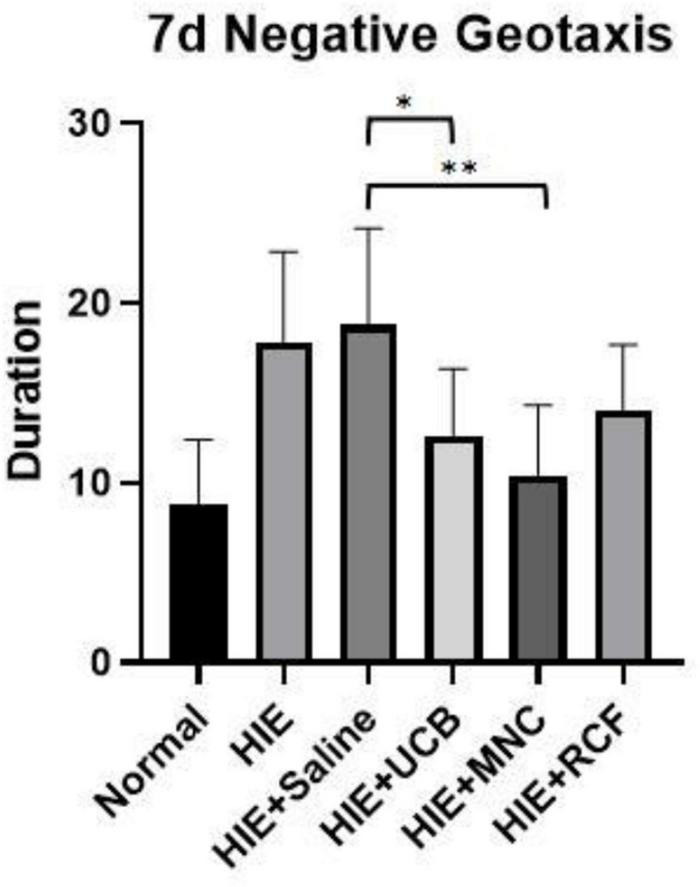
Negative geotaxis results on day 7 after cell transplantation (*n* = 8). The UCB- and MNC-treated groups showed a better performance than the saline-treated HIE and HIE-only groups. “*” Indicates *p* < 0.001, “^**^” indicates *p* < 0.0001 (one-way ANOVA with Tukey’s multiple comparison test).

In the negative geotaxis test performed on day 7 after cell transplantation (Figure 2), rat pups in the MNC-transplanted group showed a better performance than those in the HIE-only group (*p* = 0.0048), but their performance did not reach the level shown by the pups in the normal group. The performances of both the whole blood HUCB-transplanted (*p* = 0.0088) and MNCs-transplanted (*p* = 0.0001) groups were better than that of the saline-treated HIE group. However, no statistical differences in performance were found between the RCF-treated group and the saline-treated HIE (*p* = 0.3981) and HIE-only (*p* = 0.7046) groups.

### Motor Behavior Test

**FIGURE 3:**
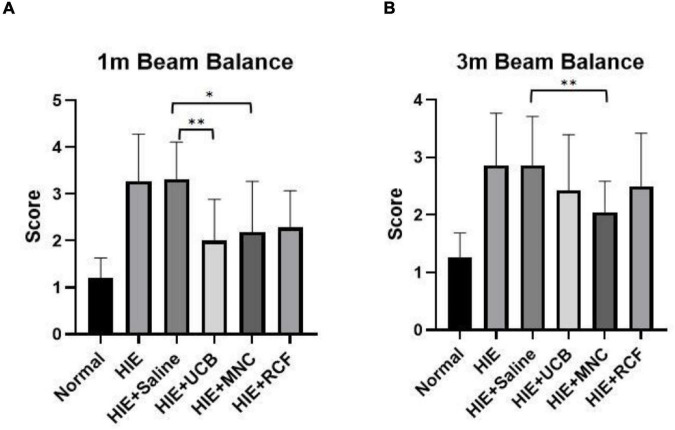
Beam balance results at **(A)** 1 month and **(B)** 3 months after cell transplantation (*n* = 12). The MNC-treated group showed better recovery of motor function than the HIE-only and saline-treated groups at both time points (1 and 3 months). “*” Indicates *p* < 0.05, “^**^” indicates *p* < 0.005 (one-way ANOVA with Tukey’s multiple comparison test).

The beam balance test was performed to assess motor behavior at 1 month (Figure 3A) and 3 months (Figure 3B) after cell transplantation. At 1 month, the UCB-treated (*p* = 0.0012), MNC-treated (*p* = 0.0119), and RCF-treated (*p* = 0.0288) groups all scored lower (indicating lesser time) than the HIE-only group, indicating that the three treated groups showed better motor balance and coordination; however, their performance did not reach the level shown by the rat pups in the normal group. Compared with the saline-treated HIE group, both the HUCB-treated (*p* = 0.0037) and MNCs-treated (*p* = 0.0245) groups (but not the RCF-treated group, *p* = 0.0514) performed better, but their performance did not reach the level shown by the normal group. At 3 months, only the MNC-treated group showed a better motor performance than the HIE-only (0.0022) and saline-treated HIE (0.002) groups, but the performance did not reach the level shown by the normal group.

### Morris Water Maze

**FIGURE 4:**
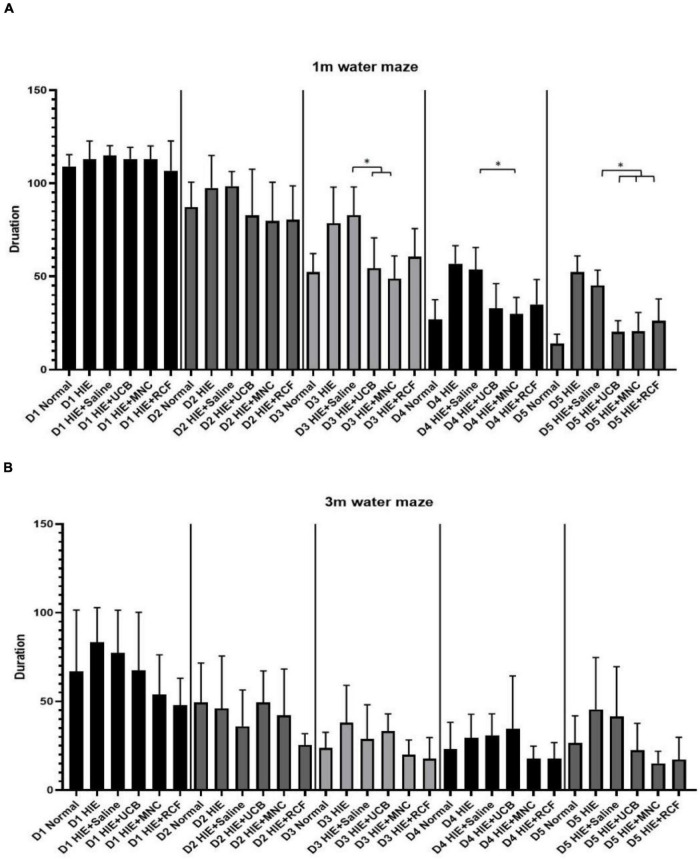
Results of Morris water maze training trials at **(A)** 1 month and **(B)** 3 months after cell transplantation (*n* = 12). On day 5 of training at 1 month, the time taken to find the escape platform was shorter in the UCB- and MNC-treated groups than in the saline-treated HIE groups. At 3 months, no difference in the water maze test performance was observed between any group, suggested the spatial learning and memory have not improved further. “*” Indicates *p* < 0.05 (one-way ANOVA with Tukey’s multiple comparison test).

The Morris water maze test (*n* = 12) was performed at 1 month (Figure 4A) and 3 months (Figure 4B) after cell transplantation to assess the spatial learning memory function of the rat pups. Five-day training trials were conducted at both time points. At 1 month, the performances of the HUCB-treated, MNCs-treated, HIE-only and saline-treated groups on day 3 were significantly different from one another, whereas on day 5, the rats in both the UCB- and MNC-treated groups found the escape platform sooner than those in the HIE-only and saline-treated groups. At 3 months, no differences were observed in the escape latency between the normal and the three treated groups. In the probe trials at 1 and 3 months, wherein the escape platform was removed, no differences were found between the groups in terms of the cumulative distance and island crossing.

### Immunohistochemical Analysis of NeuN-Positive Cells

**FIGURE 5:**
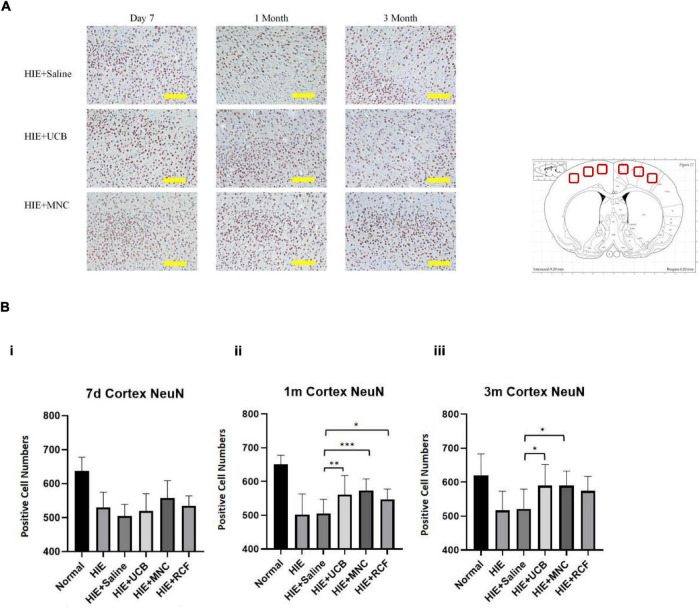
**(A)** Showed NeuN staining of control, UCB and MNC-treated rats’ motor cortex. Scale = 200 μm. **(B)** Showed NeuN-positive cell counts at 7 days, 1 and 3 months after cell transplantation (*n* = 8). More NeuN-positive cells were detected in cortex sections from HUCB and MNC-treated rats than in sections from HIE-only and saline-treated HIE rats. “*” Indicates *p* < 0.05, “^**^” indicates *p* < 0.005, “^***^” indicates *p* < 0.0001 (one-way ANOVA with Tukey’s multiple comparison test). Scale = 200 μm.

At 7 days, 1 and 3 months (Figure 5A) after cell transplantation, paraffin-embedded brain tissue sections were used for an immunohistochemistry study of neurons in the motor-sensory cortex. NeuN-positive cells were counted using software provided by Nikon. The results indicated that on day 7, all HIE groups (both with or without treatments) showed a reduced number of neurons compared with those in normal rats. Although the MNCs-treated group showed a greater number of NeuN-positive cells than the other HIE groups, the differences were not statistically significant (MNCs-treated group vs. HIE-only group, *p* = 0.818; vs. saline-treated HIE group, *p* = 0.1746).

At 1 (Figure 5B) month after cell transplantation, the UCB-treated (*p* = 0.0024), MNC-treated (*p* < 0.0001) and RCF-treated (*p* = 0.0191) groups all showed bigger numbers of neurons compared with the saline-treated HIE group. Compared with the HIE-only group, the HUCB-treated (*p* = 0.0097) and MNCs-treated (*p* < 0.0001) groups had more neurons in the motor-sensory cortex. However, no statistical difference was observed in the number of neurons between the HIE-only and RCF-treated groups (*p* = 0.0566). At 3 months (Figure 5B), both the UCB-treated (*p* = 0.0154) and MNC-treated (*p* = 0.019) groups showed more neurons compared with the saline-treated HIE group. Compared with the HIE-only group, the HUCB-treated (*p* = 0.0123) and MNCs-treated (*p* = 0.015) groups also showed more neurons in the motor-sensory cortex. Even at this time point, however, the number of neurons between the HIE-only and RCF-treated groups (*p* = 0.0759) and between the saline-treated HIE and RCF-treated groups (*p* = 0.0975) were not statistically different. In line with the results of the motor behavior tests, the cell-treated groups had more neurons in the motor-sensory cortex at both 1- and 3-month time points after cell transplantation, but these numbers did not reach those found in the normal group rats.

## Discussion

In our study, we intravenously transplanted HUCB, MNCs and the RCF into different groups of rat pups 24 h after HIE modeling. Forty-eight hours after transplantation, human Alu Yb8 expression was detected by real-time PCR to confirm the migration of a small number of the transplanted cells into the brain, contrasting previous studies in the literature (Meier et al., 2006; Yasuhara et al., 2010), where a significant number of human MNCs were present in the grafted brain. To test the short-term effects of cell therapy, the negative geotaxis test was conducted to assess neurological development on day 7. To test the long-term effects, beam balance and Morris water maze tests were performed to evaluate motor performance and spatial learning respectively, at 1 and 3 months. Furthermore, the NeuN immunohistochemistry was carried out to assess the therapeutic efficacy of HUCB and its cellular components (MNCs and RCF) in estimating the number of mature neurons in the motor-sensory cortex.

The day 7 behavioral test demonstrated that MNCs-treated HIE rats performed significantly better in the negative geotaxis suggesting a functional improvement after MNCs-treatment. At 1 month after cell injection, statistically significant differences were observed between HIE rats and HUCB, MNCs and RCF-treated HIE rats and between saline-treated HIE rats and HUCB and MNCs-treated HIE rats. At 3 months, significant differences were observed between HIE and MNCs-treated HIE rats and between saline-treated HIE rats and MNCs-treated HIE rats. These findings suggest that treatment with HUCB and MNCs led to long-term improvements in the motor function of the rat pups. The favorable treatment outcomes with MNCs are particularly consistent, for the early 7-day, and the long term one and 3 months after the intravenous administration.

The results of histological analysis of NeuN quantification at 1 month after cell injection revealed statistically significant differences between HIE rats and HUCB and MNCs-treated HIE rats and between saline-treated HIE rats and HUCB, MNCs and RCF-treated HIE rats. At 3 months after cell injection, significant differences were found between HIE rats and HUCB and MNCs-treated HIE rats and between saline-treated HIE rats and MNCs-treated HIE rats. These results indicate that MNCs transplantation increases the number of neurons in the motor-sensory cortices of HIE rats.

For this current study, there were limitations. For histology, only NeuN immunohistochemistry was employed to quantify the number of mature neurons in the sensory-motor cortex. Other important aspects such as brain water content, brain atrophy and angiogenesis were not investigated. Apoptosis and necrosis were assessed early (Lyu et al., 2021) but not long term. A systematic motor, learning and memory tests were not planned in this study. For future treatment study of HIE, for instance comparing MNCs with exosomes of HUCB, all these and the long-term behavioral tests such as gait analysis, forelimb and hind-limb function, and cylinder tests (Balkaya et al., 2018).

In this study, all HUCB and its components were transplanted to HIE rat pups immediately after processing without cryopreservation. The transportation duration was controlled to within one hour and cell viability was maintained over 80%. Weise et al. indicated that cryopreserved HUCB-derived MNCs do not perform well in the treatment of experimental focal cerebral ischemia in achieving favorable long-term neurological recovery, reducing infarct volume and brain atrophy (Weise et al., 2014).

Our findings in HIE rat pups suggest that several potential mechanisms contribute to the therapeutic effects of human HUCB-derived MNCs treatment, namely neuroprotection, neurogenesis and angiogenesis.

### Neuroprotection

On day 7 after MNC transplantation, the MNCs-treated rat pups performed better in the negative geotaxis test compared with HIE control rats. Although quantitative analyses of NeuN expression in the brain cortex showed no statistical difference between the MNC-treated and HIE groups, the average number of neurons was higher in the MNCs-treated rat cortex. These results suggest a potential neuroprotective effect against HIE within 1 week after MNC transplantation. A previous study reported a similar neuroprotective effect of MNCs in an *in vitro* cell culture experiment and suggested that multiple MNC types contribute to this effect (Reich et al., 2008).

### Neurogenesis

The quantitative analysis of NeuN expression demonstrated that the average number of cortical neurons in the HIE rat pups increased at 1 and 3 months after cell transplantation, compared with that on day 7 after cell transplantation. This suggests a potential neurogenic effect on the damaged brain areas of HIE rats. In line with this result, freshly isolated HUCB-derived MNCs have been reported to express brain-derived neurotrophic factor (BDNF), which plays a role in reducing neuronal cell death and stimulating neurogenesis, thereby enhancing the regeneration of damaged brain areas after injury (Bath and Lee, 2010; Nagahara and Tuszynski, 2011).

### Angiogenesis

One recent study has demonstrated that bone marrow derived MNCs suppressed autophagy in focal ischemia mice by activating angiogenesis through gap junction-mediated signaling. They also proved this procedure was induced by activation of hypoxia inducible factor (HIF)-1α (Kikuchi-Taura et al., 2020). As bone marrow MNCs shows a similar cell component, angiogenesis could act as another potential mechanism of HUCB-MNCs transplantation.

They further indicated that bone marrow MNCs restored cerebral metabolism after their transplantation to aging mice resulting in improve neurological functions. The underlying mechanism is related to glucose transporter expression level and activity of Na+/K+-ATPase in mice brain (Takeuchi et al., 2020).

Hypoxic-ischemic encephalopathy is a serious condition in new-born babies that may result in permanent motor dysfunction, such as CP, or even death. In recent decades, hypothermia therapy has been established to be an effective intensive care treatment modality for neonates with HIE. However, this therapy has not been able to decrease the mortality rate among HIE cases to below the 20% (Shankaran et al., 2005). Therefore, additional novel therapy development, both in mortality and disability reduction, is urgently required. Currently, many preclinical studies are investigating the efficacy of HUCB and bone marrow-derived stem cells (Nabetani et al., 2018).

In this 40-year period, a number of animal studies have suggested a few mechanisms where HUCB may exert its therapeutic effects against HIE. Five stages of the HIE processes have been identified: (1) energy consumption, (2) inflammation, (3) cellular excitotoxicity, (4) oxidative stress and (5) cell death. Specifically, HUCB treatment has been hypothesized to ameliorate inflammation and oxidative stress, preventing apoptosis and promoting brain regeneration (Epstein and McCord, 1985; Ferriero, 2004; Johnston et al., 2011).

It remains unclear which specific components of the HUCB is the major contributor to its anti-inflammatory effect in HIE treatment. Studies have shown that HUCB-derived MSCs and endothelial progenitor cells exert inflammation-ameliorating effects after brain injury. In addition, MSCs can regulate immune reactions, which may be beneficial against both local and global neuro-inflammatory processes occurring in HIE (Wasielewski et al., 2012; Rosenkranz et al., 2013; Paton et al., 2017). Some studies have demonstrated that intraperitoneal injection of HUCB-derived MNCs 6 h after ischemia could decrease cell death and free radicals (Rosenkranz et al., 2012; Hattori et al., 2015). Another type of human-derived cells, such as CD34^+^ hematopoietic stem cells, have been shown to secrete numerous growth factors, including BDNF, glial cell-derived neurotrophic factor and vascular endothelial growth factor (VEGF), all of which could promote recovery from brain damage in HIE (Yoshihara et al., 2008; Rosenkranz et al., 2010). CD34^+^ cells have also been reported to provide an environment beneficial for neuronal regeneration after ischemic stroke (Taguchi et al., 2004a,b). In our study, based on real-time PCR results, organs like liver, lung and heart harvested large amounts of injected cells, these cells may also have the possibility of releasing cytokines, growth factors, neurotrophic factors or other small molecules. These factors may enter the brain through an impaired blood-brain barrier (BBB).

Human umbilical cord blood is a well-known source of a variety of stem cells, including CD34^+^ cells and endothelial progenitor cells. The proportion of CD34^+^ cells in HUCB is approximately 1–2%, in comparison with less than 0.01% in adult human peripheral blood (Murohara et al., 2000; Rafii and Lyden, 2003; Sun et al., 2010). HUCB treatments have been shown promising effects against hematological diseases, including leukemia, and this treatment method has replaced the use of hematopoietic stem cells in the last few decades (Tezuka et al., 2002; Gluckman and Rocha, 2004). Furthermore, as a source of endothelial stem cells, HUCB has shown therapeutic effects against many diseases, such as CP, diabetes and cardiovascular diseases (Lindvall and Kokaia, 2006).

Many studies have focused on the use of MNCs to treat HIE. Sharma et al. (2015) reported that bone marrow-derived MNC therapy led to behavioral improvements in patients with CP at the 6-month follow-up. Nuclear Medicine Imaging showed improved metabolism in damaged brain areas in these patients, which were consistent with the observed clinical improvements. Based on these results, the researchers concluded that MNC therapy can stimulate neurological development, reduce brain dysfunction and promote the quality of life in patients with CP (Sharma et al., 2015).

Translating bench research findings into clinical trials in a timely and methodologically appropriate manner, it is logical to follow the adult stroke community’s STEP 4 movement, using stem cells therapy as an emerging paradigm to advance and to accelerate preclinical research (Boltze et al., 2019) as HIE is a form of neonatal ischemia. A recent example of the timing of specific and intensive stroke rehabilitation directly translated from rodent experiments to human, showing rehabilitation early within 30 days and late beyond 6 months are not effective. The most effective period has been proven to be 60–90 days (Dromerick et al., 2021). Our experimental work on HUCB and its MNCs treating HIE can be fast-tracked to clinical trials when its availability, dosage and timing are worked out.

## Conclusion

Our data suggest that

(1) HUCB transplantation can improve short-term and partially long-term neurological function in neonatal rats with HIE.
(2) RCF has a partial long-term treatment effect in neonatal rats with HIE.
(3) HUCB-derived MNC is an effective therapy that can improve both short-term and long-term neurological function in neonatal rats with HIE.

## Data Availability Statement

The original contributions presented in the study are included in the article/supplementary material, further inquiries can be directed to the corresponding author/s.

## Ethics Statement

The animal study was reviewed and approved by the Ethics Committee of The Chinese University of Hong Kong.

## Author Contributions

WP, WY, and CW conceived and designed the study. HL contributed to the animal experiments, behavioral tests, histology and statistical analysis. JC and YH contributed to the animal observation and care. SL and ZZ contributed to the cell quantification. DS, CN, and GH contributed to the technical support. WP, WY, CW, DS, and CN supervised the study. HL drafted the manuscript. WP, CN, and WY revised the manuscript. All authors contributed to the article and approved the submitted version.

## Conflict of Interest

WC was employed by the company Mononuclear Therapeutics Limited. The remaining authors declare that the research was conducted in the absence of any commercial or financial relationships that could be construed as a potential conflict of interest.

## Publisher’s Note

All claims expressed in this article are solely those of the authors and do not necessarily represent those of their affiliated organizations, or those of the publisher, the editors and the reviewers. Any product that may be evaluated in this article, or claim that may be made by its manufacturer, is not guaranteed or endorsed by the publisher.

## Footnotes

1
https://apps.thermofisher.com/apps/spa/#/dashboard

## Abbreviations

HIE: hypoxic-ischemic encephalopathy
HUCB: human umbilical cord blood
MNC: mononuclear cells
RCF: red cell fraction
CP: cerebral palsy
CBF: cerebral blood flow
MSC: mesenchymal stem cells
CCA: common carotid arteries.
